# Neural Networks-Based On-Site Dermatologic Diagnosis through Hyperspectral Epidermal Images

**DOI:** 10.3390/s22197139

**Published:** 2022-09-21

**Authors:** Marco La Salvia, Emanuele Torti, Raquel Leon, Himar Fabelo, Samuel Ortega, Francisco Balea-Fernandez, Beatriz Martinez-Vega, Irene Castaño, Pablo Almeida, Gregorio Carretero, Javier A. Hernandez, Gustavo M. Callico, Francesco Leporati

**Affiliations:** 1 Department of Electrical, Computer and Biomedical Engineering, University of Pavia, 27100 Pavia, Italy; 2 Research Institute for Applied Microelectronics (IUMA), University of Las Palmas de Gran Canaria (ULPGC), 35001 Las Palmas de Gran Canaria, Spain; 3 Norwegian Institute of Food, Fisheries and Aquaculture Research (Nofima), 6122 Tromsø, Norway; 4 Department of Psychology, Sociology and Social Work, University of Las Palmas de Gran Canaria, 35001 Las Palmas de Gran Canaria, Spain; 5 Department of Dermatology, Hospital Universitario de Gran Canaria Doctor Negrín, Barranco de la Ballena, s/n, 35010 Las Palmas de Gran Canaria, Spain; 6 Department of Dermatology, Complejo Hospitalario Universitario Insular-Materno Infantil, Avenida Maritima del Sur, s/n, 35016 Las Palmas de Gran Canaria, Spain

**Keywords:** skin cancer, hyperspectral imaging, deep learning, disease diagnosis, high-performance computing

## Abstract

Cancer originates from the uncontrolled growth of healthy cells into a mass. Chromophores, such as hemoglobin and melanin, characterize skin spectral properties, allowing the classification of lesions into different etiologies. Hyperspectral imaging systems gather skin-reflected and transmitted light into several wavelength ranges of the electromagnetic spectrum, enabling potential skin-lesion differentiation through machine learning algorithms. Challenged by data availability and tiny inter and intra-tumoral variability, here we introduce a pipeline based on deep neural networks to diagnose hyperspectral skin cancer images, targeting a handheld device equipped with a low-power graphical processing unit for routine clinical testing. Enhanced by data augmentation, transfer learning, and hyperparameter tuning, the proposed architectures aim to meet and improve the well-known dermatologist-level detection performances concerning both benign-malignant and multiclass classification tasks, being able to diagnose hyperspectral data considering real-time constraints. Experiments show 87% sensitivity and 88% specificity for benign-malignant classification and specificity above 80% for the multiclass scenario. AUC measurements suggest classification performance improvement above 90% with adequate thresholding. Concerning binary segmentation, we measured skin DICE and IOU higher than 90%. We estimated 1.21 s, at most, consuming 5 Watts to segment the epidermal lesions with the U-Net++ architecture, meeting the imposed time limit. Hence, we can diagnose hyperspectral epidermal data assuming real-time constraints.

## 1. Introduction

Preceded by several other tumors in population incidence, skin cancer affects the largest organ in the body and represents one of the most frequent malignancies [1]. Physicians usually distribute epidermal lesions into two etiologies, namely melanoma and non-melanoma skin cancer (NMSC). Most skin cancers begin in the epidermis and can affect three types of cells: squamous cells, basal cells, or melanocytes. The MSC originates from any cell capable of forming melanin, and is divided into three subtypes, namely superficial extension, lentigo maligna, and nodular [2]. Some types of skin cancer present genetic modifications that, if left untreated, grow, and spread over the body, inducing potentially metastasizing conditions.

Although MSC is the rarest skin tumor, it, nevertheless, causes the highest mortality rates because of an absence of adequate early detection. NMSC lesions represent more than 98% of the known skin lesions in the United States of America, of which 75–80% are basal cell carcinoma (BCC), 15–20% are squamous cell carcinoma (SCC), and around 1.6% is MSC, the most lethal type of cancer [3]. However, BCC and SCC must be considered malignant as they might degenerate into malignancies and induce death [4]. Therefore, sorting epidermal tumors into benign and malignant categories is more accurate. Currently, a person has a 4% chance of developing melanoma, which is responsible for 75% of all skin cancer-related deaths [1,5,6].

In routine clinical diagnosis, dermatologists perform a visual inspection of melanocytic tumors to determine the presence of malignancies; they employ a handheld device that comprises magnifying lenses and uniform polarized illumination. The examination procedure relies upon the so-called ABCDE rule, where A stands for asymmetry, B for border irregularity, C for color, D for diameter, and E for evolution [7]. However, this procedure introduces false positives, namely benign lesions classified as malignant. Hence, the gold standard consists of a biopsy that requires surgical excision of the lesion and histopathological inspection [7,8]. Nevertheless, this process is not only painful, but also time-consuming, slow, and expensive [5]. Indeed, the worldwide incidence of skin cancer is still rapidly rising, bearing a heavy health and economic burden for diagnosis and treatment. Early detection of skin cancer effectively improves the 5-year survival rate and is correlated with 99% of the overall healing probability. Hence, the escalating rate of skin cancers, and the lack of adequate expertise and innovative methodologies present an immediate demand for systems based on artificial intelligence (AI) and novel optical technologies to assist clinicians in this domain [7,9].

In this context, hyperspectral imaging (HSI) is a non-invasive, non-ionizing, and label-free technique, originally created for remote-sensing and military purposes, that is becoming more popular in medicine for cancer detection thanks to recent technological advances [7,10]. Hyperspectral (HS) images measure the reflected or transmitted light from the captured scene, collecting light–matter interaction values associated with several wavelengths of the electromagnetic spectrum range with low to high spatial resolution. HS images comprise multiple images aligned in adjacent narrow wavelengths, forming a reflectance spectrum of all the pixels [11,12,13,14]. Thus, the outcome is a HS cube, which contains both the spatial and the spectral information from the sample under analysis. 

Chromophores, such as melanin and hemoglobin, are organic molecules which characterize the spectral properties of epidermal lesions and vary among skin lesions of diverse etiologies. Consequently, HSI systems should capture such information, enabling the use of machine learning (ML) algorithms to automatically detect and cluster tumors of various categories [7,11,15]. Traditional imaging techniques are limited to the visible light spectrum, leading to limited diagnostic results. However, HS images set the stage for broadband information acquisition, overcoming inter-class similarities and intra-class dissimilarities of various diseases considered in the visual domain [9,10,11].

Conceived to detect skin cancer at the early stages, researchers aimed to design AI solutions to strengthen current diagnostic performances whose effectiveness relies heavily upon dermatologist expertise [16,17,18]. Several research reviews considered learning-based studies concerning skin cancer diagnosis adopting several types of data, including HS images, highlighting their strengths and weaknesses. In particular, authors of different systematic review articles focused on more than fifty investigations concerning different data types and learning methodologies, involving hundreds of dermatologists for direct comparison [5,6,8,9]. 

Furthermore, research should not be limited to the learning system but also to designing a device to overcome current challenges, such as data availability, interpretability, and computational power, employing recent algorithms and having real-world clinical scenario applicability. Indeed, although current AI algorithms are still at the very early stages of clinical application and not always ready to aid clinicians, they can be scalable to multiple devices, transforming them into modern medical instruments [9,19]. Such novel devices will also store the acquired data, overcoming the data availability issues.

Present solutions differ mainly in the architectures employed, namely artificial neural networks (ANNs) and convolutional neural networks (CNNs), and the data used for training. Indeed, most investigations employed CNNs and dermoscopic images to diagnose epidermal lesions, since deep learning (DL) algorithms and high-quality data grant a significant level of performance. At first, researchers used ANNs to replicate the ABCD methodology with accuracy levels between 70 and 90%. However, small-diameter lesions made the diagnosis more demanding, causing the algorithm to introduce misclassifications [5,6]. Although researchers reduced the problem by introducing CNNs, the difficulty remains, since lesions from different etiologies have subtle visual variations. In general, most investigations feature diagnostic performances comparable to expert dermatologists, whose sensitivity, specificity, and accuracy concerning benign and malignant lesions are acknowledged to be around 80, 75, and 70% [6,20]. Board-certified dermatologist accuracy decreases to around 55% when more classes are considered for diagnostic relevance [20]. Hence, considering multi-class classification scenarios, diagnostic evaluation measurements are worse. Furthermore, not only did studies show that low specificity was usually traded off for high sensitivity, but also that metrics were typically biased due to the lesions considered already being marked as suspicious prior to investigation [6,17,18,20,21]. Indeed, results show that DL algorithm performance improved over 90% only when researchers conducted experiments with an unconventional binary classification task, namely malignant melanoma (MM) and BCC or nevus [5]. Other studies involved histopathological and clinical images. The early studies exhibited comparable performance concerning dermoscopic data. However, pathologists surgically removed part of a suspicious lesion and applied labelling to conduct a diagnosis. Besides, clinical images presented worse diagnostic evaluations, made worse still when the researchers considered more than two etiologies [6,21].

**Figure 1:**
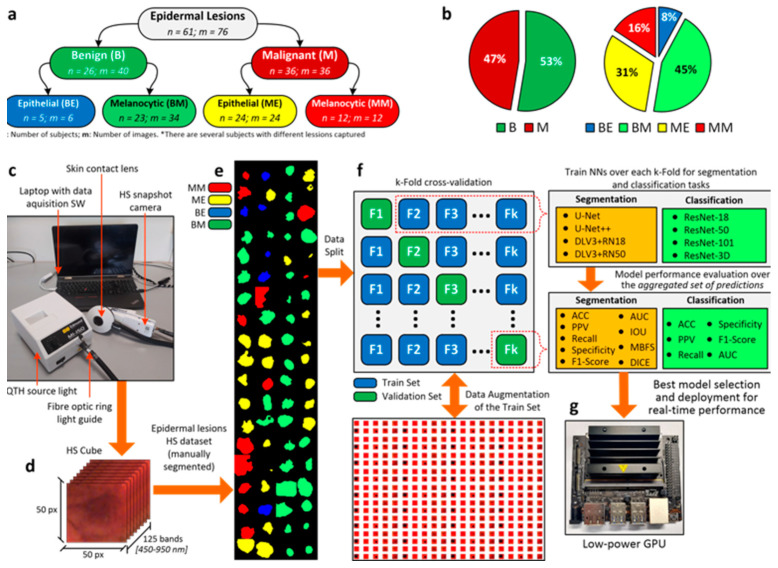
Proposed experimental framework. (**a**) Taxonomy of the epidermal lesions included in the HS database, including number of subjects and images in each category; (**b**) Distribution of images for the binary (left) and multilabel (right) classification problems; (**c**) Different elements of the HS acquisition system; (**d**) HS cube characteristics; (**e**) HS dataset ground-truths; (**f**) Proposed processing framework based on a k-fold cross-validation, including data augmentation and aggregated model evaluation; (**g**) Low-power Nvidia Jetson GPU for algorithm deployment to reach real-time performance.

The main contribution of our work is the proposal of a DL pipeline comprising eight different architectures for the classification and segmentation of HS in vivo skin lesion images (Figure 1). Enhanced by data augmentation, transfer learning, and extensive hyperparameter tuning, we trained the networks with a database composed of 76 HS epidermal lesions from 61 patients [14]. Pathologists and dermatologists diagnosed suspected malignant lesions through biopsy-proven histological assessment to evaluate the tumor etiology, categorizing each lesion in the proposed taxonomy (Figure 1a,b). Data were captured using a customized HS acquisition system [14] (Figure 1c) and segmentation masks originated from the HS cubes (Figure 1d) to delimit the lesion boundaries in the images (Figure 1e). In particular, dermatologists manually segmented the boundaries of the lesions in the database. Different ML algorithms were previously proposed [14], whose outcomes encouraged the introduction of improved developments from a set of CNNs trained to identify, segment, and classify epidermal lesions in, at most, four categories following a k-fold cross-validation approach (Figure 1f). In this work, we also provide a lesion-border segmentation map. Researchers highlighted the lack of semantic information provided to physicians. Indeed, identifying the lesion boundary could lead to decreasing the chances of lesion reoccurrence and to an increased healing probability [9]. Moreover, we deployed a semantic segmentation network in a portable device [14,22] equipped with a low-power graphics processing unit (GPU), targeting routine clinical testing (Figure 1g). We responded to the demand for an AI pipeline to fit a real-world clinical scenario, which could assist dermatologists to scale up skin cancer screening and early detection, reducing the number of false positives and negatives and, hence, the number of biopsies and histopathological evaluations [9]. Recent studies [18,23] presented promising results of AI applications in various domains, again highlighting the lack of adequate computing power to process DL algorithms [5,19]. The proposed architectures, targeting handheld medical instrument deployment, attained and enhanced the well-known dermatologist human-level detection performances for both malignant-benign and multilabel classification tasks, as they were able to diagnose HS data considering real-time constraints for on-site diagnostic examinations.

## 2. Materials and Methods

### 2.1. HS Dermatologic Acquisition System

A custom solution to acquire HS epidermal lesions was developed [14,22]. The system was composed of a snapshot camera (Cubert UHD 185, Cubert GmbH, Ulm, Germany) capable of capturing the visual and near-infrared (VNIR) spectrum. The captured spectral range covered from 450 to 950 nm, bearing a spectral resolution of 8 nm and a spatial resolution of 50 × 50 pixels, whose pixel size was 240 × 240 µm^2^. The camera was coupled with a Cinegon 1.9/10 lens (Schneider Optics Inc., Hauppauge, NY, USA) with a 10.4 nm focal length. The acquisition system employed a Dolan-Jenner halogen source light (Dolan-Jenner, Boxborough, MA, USA) and the lamp employed was a 150 W quartz-tungsten bulb. A fiber optic ring light guide was coupled to the HS camera and employed to illuminate the skin surface with cold light, avoiding the high temperature of a halogen lamp on the subject skin. A dermoscopic lens with a human skin refraction index was embedded in a 3D-printed contact structure and attached to the system. The system allowed the capture of HS images in 250 ms. Finally, the system was connected to a laptop to be controlled by the acquisition software.

### 2.2. Dataset

The data acquisition campaign was carried out from March 2018 to June 2019 at two hospitals: Hospital Universitario de Gran Canaria Doctor Negrín (Canary Islands, Spain) and the Complejo Hospitalario Universitario Insular-Materno Infantil (Canary Islands, Spain). The database was composed of 76 HS images, 40 benign and 36 malignant skin lesions, from 61 subjects [14]. Pathologists and dermatologists diagnosed suspected malignant lesions through biopsy-proven histological assessment to evaluate the tumor etiology, categorizing each lesion in the taxonomy described in Section 2.3. Supplementary Table S1 describes the dataset in detail.

### 2.3. Epidermal Lesion Taxonomy

We arranged epidermal lesions in a tree structure with two root nodes representing broad disease classes, namely benign and malignant lesions. Only one other tree level besides the main root was considered. Specifically, the researchers split each root node into melanocytic and epidermal tumors [21] (Figure 1a). This taxonomy was adopted as a trade-off between other classification approaches, introduced as it was medically relevant, complete, and well-suited to ML classifiers. Our taxonomy is well-suited to treat patients according to the highest healthcare standards and provides the best classification performance. On the one hand, the root layer nodes are used in the first validation approach and represent the broadest partition. On the other hand, the children layer represents disease classes sharing similar clinical treatment procedures. Consequently, dermatologists are able to diagnose more severe lesions earlier and improve patient survival rates [21]. Pathologists and dermatologists diagnosed suspected malignant lesions through biopsy-proven histological assessment to evaluate the tumor etiology. They assigned each epidermal lesion a category from the taxonomy proposed. Additionally, a mask highlighting the tumor borders was performed by visual inspection of the synthetic RGB images generated from the HS cubes.

### 2.4. Use of Human Subjects

Board-certified dermatologists performed the acquisition campaign under informed consent. The “Comité Ético de Investigación Clínica-Comité de Ética en la Investigación (CEIC/CEI)”, from both the hospitals involved in our research, approved both the study protocol and the consent procedures.

### 2.5. Data Pre-processing

HS data pre-processing was performed to standardize the spectral signature among different patients and acquisitions due to possible variations in illumination conditions [12,14,22]. First, two reference images were captured before recording the skin lesions: a white reference image (WI) was acquired, captured from a white reference tile able to reflect 99% of the incident light, and a dark reference image (DI) was recorded when the light was turned off and the camera shutter was closed. Hence, the calibration of the raw HS image (RI) was performed following Equation (1), where CI is the calibrated image.

(1) $$CI=\frac{RI-DI}{WI-DI}$$

Second, we reduced the spectral noise in the HS data by removing the first four and the last five bands due to the poor response of the HS sensor in the extreme bands. Moreover, we used a smoothing filter based on a moving average algorithm with a window of 5. Additionally, each spectral signature was normalized into the range [0, 1] using the min–max procedure. The final spectral signature contained 116 wavelengths.

The pre-processing stage ensured the comparison of illuminating conditions, allowing DL algorithms to focus on the spectral signature shape. The authors assessed the integrity of acquisitions, performing repeated experiments and ensuring that data did not change between recordings.

### 2.6. Deep Learning Methodology

Eight CNNs architectures were trained to both classify and perform the semantic segmentation of the HS skin lesion images. On the one hand, ResNet-18, ResNet-50, ResNet-101, and a ResNet-50 variant, which exploits 3D convolutions [24], were employed to classify the images into the taxonomy presented in the previous section. On the other hand, U-Net [25], U-Net++ [26], and two versions of the DeepLabV3+ architecture [27]—one having as backbone structure a ResNet-18 and the other a ResNet-50—were evaluated to perform semantic segmentation of the epidermal lesions. Furthermore, a commonly known procedure, transfer learning [28], was adopted to improve the results of the learning-based architecture by exploiting features belonging to the previous training task. The best common practice suggests using neural networks architectures, pre-trained on similar domains, to overcome small-sized dataset problems and poor performances. Therefore, all the listed architectures had already undergone optimization based on the ImageNet dataset [29]. Particularly, MATLAB offers the possibility of instantiating already-trained deep learning models which can be modified to accept different image sizes.

The training set statistical assortment was increased by applying data augmentation to the HS images using several diversifications, including geometric (i.e., rotation, mirroring, scaling, cropping etc.), filtering, random center cropping, color transformations, and pixel substitution. We performed either a linear combination of random pixels of tumors belonging to the same category, or directly exchanged them. The same procedure was applied to skin pixels. We finally obtained approximately ten thousand images are the training set.

Data augmentation produces effective results in computer vision tasks, significantly reducing overfitting [30]. Furthermore, we introduced salt-and-pepper white noise in random image bands to enlarge the training set. The augmentation procedure was carried out iteratively. One of the data augmentation techniques was applied to the training set. A new data cluster was then created by unifying the original images and the transformed images. Following this, a second technique was applied to the new group. Finally, this procedure was recursively applied to broaden the training set exponentially. Such augmentation techniques were not applied to either the validation or the test sets, to prevent the results being biased.

All architectures were modified to receive input size 50 × 50 × 116, concerning height, width, and number of bands. We not only placed a dropout layer in each ResNet architecture, but we also introduced the L2 weights penalty in the loss function to additionally reduce overfitting. The semantic segmentation networks already met the requirement in their original design. Cross-entropy loss function and the Adam method [31] were used for training. The learning step was reduced by multiplying it by the dropping factor: it steadily and linearly decreased after each predetermined number of epochs. Batch size, number of epochs, learning rate, and drop factor period were set to 32, 10, $9\times 10^{-5}$, and 3, respectively, for all architectures. The drop factor and L2 penalty were set to $5\times 10^{-1}$ and $10^{-4}$, respectively, for the semantic segmentation models, and to $10^{-2}$ and $9\times 10^{-2}$, respectively, for the classification models.

The test system used to conduct our experiments was equipped with an Intel-i9-9900X CPU, working at 3.5 GHz, 128 GB of RAM, and two 2944-core NVIDIA RTX 2080. MathWorks MATLAB 2021b Release—Deep Learning Toolbox was used for the network design and implementation.

### 2.7. K-Fold Cross-Validation and Aggregated Validations

Cross-validation is a resampling procedure usually employed to evaluate DL models based on a limited data sample. This is a statistical method whose results in metrics estimations offer lower bias than other methods. The procedure has a single parameter called k, which refers to the number of groups in which the data sample is split. When k is as big as the data sample size, the procedure is called leave-one-out cross-validation. As such, the process is often called k-fold cross-validation. The cross-validation technique is primarily used in applied ML to estimate the performance of a model on unseen data, and was not used during the model training.

We randomly shuffled the original HS dataset comprising 76 images and split it into k groups. Specifically, we set k = 10. Next, each unique group was selected as test data and the model was trained on the remaining groups. Hence, we applied data augmentation onto the groups used for training. The model was fit on the training set and evaluated on the test set, retaining the prediction evaluated at each iteration and discarding the model. Therefore, we trained the model k times and recorded its estimate for each test set. Hence, the performance metrics for both classification and semantic segmentation were assessed on the aggregated group of predictions, namely the union of each k-fold test set, generated for each DL architecture through the procedure.

### 2.8. Performance Evaluation

We computed the occurrence of true-positive (TP), true-negative (TN), false-positive (FP), and false-negative (FN) values to evaluate the DL architectures’ performance. Concerning the semantic segmentation task, we assessed the pixel-based occurrences. The assessment outcomes were exploited to compute the following metrics: accuracy, sensitivity, specificity, precision, Receiver Operating Characteristic Area Under the Curve (AUC), precision, and F1-Score. For the segmentation task, we also computed the Mean Boundary-F1 Score (MBFS), the Intersection Over Union (IOU), and the DICE coefficient [32,33]. These metrics were evaluated over the aggregated prediction set of each architecture, which we conveyed through the k-fold cross-validation strategy. The Supplementary Methods SM presents a more detailed description of these metrics. Furthermore, the GPU accelerated-computing performance was estimated assessing the elapsed time, measured in seconds (s), and power dissipated, measured in Watts (W).

### 2.9. Architecture Selection for GPU Deployment

Each architecture’s semantic segmentation performance was evaluated. Consequently, the model having the best predictive capabilities was selected, i.e., the U-Net++. Having chosen the best model, a custom C/CUDA code was developed in terms of both the architecture’s weight and the HS epidermal lesion classification. This first serial stage ended with image pre-processing. The subsequent stage consisted of parallel semantic inference, exploiting the U-Net++ layers. The choice of having a hybrid C/CUDA code proved itself valid concerning the real-time classification of skin cancer HS images [12]. U-Net++ was a 130 layer-wise network having 130 M parameters.

### 2.10. High-Performance Computing

Several researchers stated the problem of engineering an AI-based pipeline to improve the accessibility of skin-lesion screening at the global expert level. Not only should the system be able to meet board-certified dermatologist classification performance, but it should also feature a semantic segmentation both to provide knowledge and to determine the tumor boundaries, thus improving remission and avoiding its reoccurrence [19]. Furthermore, a GPU could play a crucial role in AI-based systems for healthcare. CNNs consist of millions of parameters arranged in a matrix manner across their layers, whose multiplication with input data allows neurons to fire and highlight features to determine the diagnostic outcome. Hence, DL models can be computationally expensive. GPU deployment not only enables high-performance parallel computing, but also opens the possibility of deploying the diagnostic model on handheld devices [9,19].

Therefore, the CUDA extension to C language was employed, and a custom code to embed the U-Net++ inside a low-power NVIDIA Jetson GPU was developed. The Jetson board is a 128-core Maxwell architecture, designed for embedded applications and equipped with a quad-core ARM A57 running at 1.43 GHz. The board runs applications consuming 5 or 10 W, depending on the power budget mode set on the device. CUBLAS and cuDNN libraries were extensively used: these contain computationally efficient functions for linear algebra operations and procedures concerning DL, such as convolutional and normalization layers, activation functions, and feedforward inference. The functions operate on tensors having the following shape: number of examples (N), number of channels (C), height (H), and width (W). The C/CUDA codes were compared to the previously developed MATLAB codes at each U-Net++ building stage. Each intermediate result of the inference pipeline was evaluated and verified.

**Figure 2:**
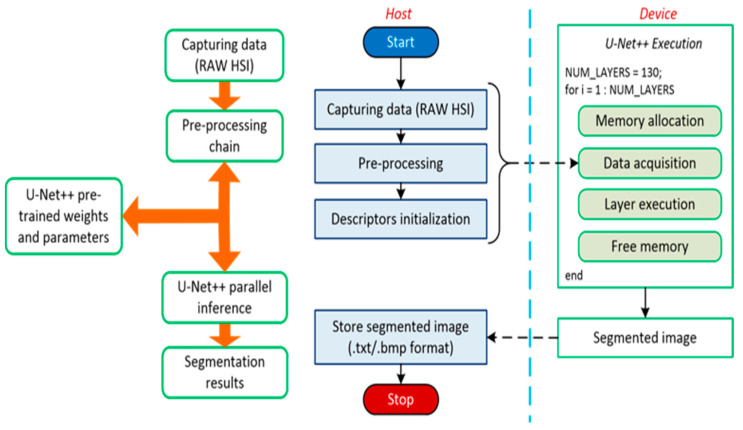
CUDA execution logic and data transfer flow.

Figure 2 shows the outcome of the custom development. Code execution starts on the CPU, the Host. The HS epidermal lesion image was captured, and the neural network weights acquired. Once all the necessary elements and descriptors were initialized, we moved to the device memory, namely the GPU memory, the data needed from the U-Net++ for inference. Due to the limited memory of the Jetson GPU, we arranged the prediction to compute a layer output at that time. Specifically, we allocated memory to each layer, acquired the previous dataflow outcome, executed the layer, produced the new result, and finally, we freed the memory. Once the loop ended, a segmented image was obtained, which we moved back to the Host, where the result was saved and displayed on the handheld device. The semantic segmentation of HS skin cancer images is performed in less than a second.

## 3. Results

### 3.1. Classification of Epidermal Lesions

The CNNs were trained and fine-tuned with a small-sized dataset. We then evaluated the performance of the architectures, employing a k-fold cross-validation methodology with k set at 10. Moreover, the taxonomy proposed in Figure 1a was adopted as a trade-off between being medically relevant, complete, and well-suited to DL classifiers. Indeed, the considered tree-structure categorization was well-suited to treat patients according to the highest healthcare standards and provided the best achievable classification performance [21]. Indeed, not only do we propose malignant-benign classifications, but also a fine-grained classification, allowing expert professionals to differentiate between numerous severe conditions.

**Figure 3:**
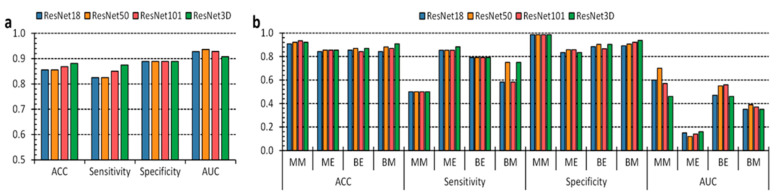
Performance of the epidermal lesion classification. (**a**,**b**), Binary and multilabel classification performance of the four different approaches, respectively.

Discrimination between benign and malignant lesions offered stable and robust measurements, meeting sensitivity and specificity above 80% (Figure 3a and Supplementary Table S2). We observed the ResNet3D achieving the best results (87% sensitivity and 88% specificity). Furthermore, we can determine through AUC outcomes that the performance could be increased by over 90% using adequate thresholding. On the other hand, the multilabel classification retains an MM, BE, and BM sensitivity performance below 80%. Nonetheless, the specificity for all classes is above 80% (Figure 3b and Supplementary Table S3). Considering that having more groups induces each group to have fewer examples, the diminished number of images in the BE and ME categories elicits sparse information regarding inter-patient variability. The multilabel classification scenario demonstrated the ResNet50 and the ResNet3D as having the best performances. A drawback of considering an aggregated validation set, i.e., the union of each k-fold test set, is the risk of having inconsistent AUC results. Indeed, researchers usually compute AUC over a single classifier, whose prediction probability retains a classification. Aggregating the results means we unify possibly disharmonious likelihoods from different classifiers trained at each k-fold iteration. That is why we observed acceptable classification metrics measurements related to the low AUC.

### 3.2. Anatomical Segmentation of Epidermal Lesions

Tumor border detection is a crucial step towards patient healing and disease remission. Not only is this step significant for skin cancer, but it also gains relevance when experts consider other tumor types, such as brain cancer [15,18,34,35,36]. Indeed, the harder the disease is to reach inside the human body, the better its boundary detection should be to avoid its reoccurrence and enhance remission probabilities. Several researchers stated the problem of engineering an AI-based pipeline to improve the accessibility of skin-lesion screening at the global expert level [5,6,17,18]. The system should be able to meet board-certified dermatologist classification performance. Furthermore, it should also comprise a semantic segmentation to provide knowledge and determine the tumor boundaries [19]. We trained the U-Net, the U-Net++, and two DeepLabV3+ versions, having ResNet-18 and ResNet-50, respectively, as the backbone structure, to answer the demand of semantic information concerning the skin lesion boundaries. We evaluated each semantic architecture through nine metrics per class considered (Supplementary Tables S4 and S5). These results also include the skin class for the computation of the results. Concerning binary segmentation (Figure 3a), we measured skin DICE and IOU higher than 0.9, apart from the DLV3 + RN18 architecture, which yielded a lower segmentation performance. Nonetheless, we observed limited performance regarding benign and malignant classes. Specifically, DICE measurements below 0.8 and IOU under 0.6. The U-Net++ exhibited the best segmentation results over all the categories. Similarly, the U-Net++ offered the best outcomes concerning the multilabel segmentation scenario (Figure 3b). However, the IOU measurements for the ME and BE categories were lower than 0.4. The results might be due to the high inter-patient variability concerning lesions etiologies and the few samples belonging to different groups. Poor MBFS measurements both in binary and multilabel segmentation tasks will be addressed in a later section.

### 3.3. U-Net++ Results and Rationale

**Figure 4:**
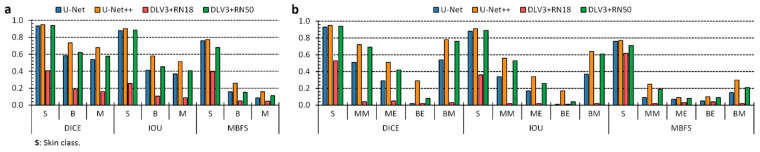
Performance of the epidermal lesion segmentation. (**a**,**b**), Binary and multilabel segmentation performance of the four different approaches, respectively. The acronyms have the following meanings: Skin (S), Benign (B), Malignant (M), Benign Epithelial (BE), Benign Melanocytic (BM), Malignant Epithelial (ME), and Malignant Melanocytic (MM).

We evaluated the U-Net++ architecture for embedded system deployment for two main reasons. First, it exhibited the best performance both in the multilabel and binary segmentation assignments (Figure 4 and Supplementary Tables S4 and S5). Furthermore, the architecture presents the highest number of layers and parameters. In other words, it is able to satisfy real-time constraints [12] with such architecture, and strongly ensures that the same time-limitation could be satisfied with smaller CNNs which will be considered in future work. Researchers define a real-time constraint as a mandatory temporal deadline to carry out a task [12]. A reasonable time limit for skin cancer detection and segmentation can be set around a few minutes since its evolution takes several weeks to elapse. We chose U-Net++ as the network for embedded deployment, and a real-time constraint set for epidermal lesion classification and segmentation was met: recording time stamps ranging from 0.230 to 1.210 s concerning different GPU architectures, which were compared in terms of time and power consumption (Figure 4).

### 3.4. U-Net++ Embedded Deployment

**Figure 5:**
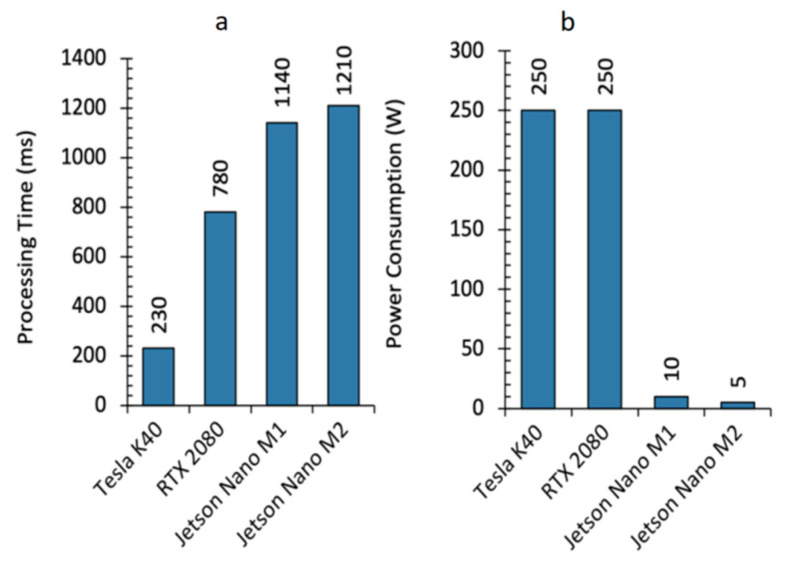
Deployment performance. (**a**) Processing time and (**b**), power consumption comparisons of the different NVIDIA GPUs considered in this study. Jetson Nano M1 and Jetson Nano M2 indicate the two possible power configurations of the Jetson Nano board, which are 10 and 5 W of power budget, respectively.

We developed a custom code through the CUDA extension to C language to embed the U-Net++ inside a low-power NVIDIA Jetson GPU. The Jetson board was a 128-core Maxwell architecture designed for embedded applications and equipped with a quad-core ARM A57 running at 1.43 GHz. The board runs applications consuming 10 or 5 W, depending on the power budget set on the device. We extensively used CUBLAS and cuDNN libraries, which contain computationally efficient functions for linear algebra operations and procedures concerning DL, such as convolutional and normalization layers, activation functions, and feedforward inference. The code was tested on three different GPU boards produced by NVIDIA. The three boards were chosen to cover the range of products proposed by this vendor. The RTX 2080 is a consumer board featuring 2944 cores working at 1.8 GHz and equipped with 8 GB of DDR6 memory. The Tesla K40 GPU is a board developed for computationally intensive applications; it is equipped with 2880 cores working at 750 MHz and 12 GB of DDR5 memory. The Jetson Nano board is a low power GPU featuring 128 cores working at 1.6 GHz and 4 GB of DDR4 memory. The Tesla K40 and RTX2080 obtained the best performance in terms of processing times (Figure 5a), with elaborations ranging from 230 to 780 ms and a power consumption of 250 W (Figure 5b). On the other hand, the Jetson Nano board took from 1.14 s to 1.21 s to processes the images, consuming 10 to 5 W (Figure 5a,b). Thus, all three boards feature processing times which would well fit the target application. Moreover, the Jetson Nano board has a power consumption which enables the development of a portable and handheld diagnostic instrument, especially the M2 power configuration.

### 3.5. Comparison with Expert Dermatologists

Researchers believe it to be difficult, and not reasonably fair, to compare studies due to different settings from data employed to algorithm structures and the proposed taxonomy. In general, HS images contain broader information that is not comparable with classical RGB pictures. Several reviews evaluated more than fifty studies retaining the different settings discussed and whose research involved hundreds of expert dermatologists [5,6,7]. We can, therefore, establish a well-known plateau of performance. Considering the proposed taxonomy, expert dermatologist sensitivity, specificity, and accuracy concerning benign and malignant lesions lie at approximately 80%, 75%, and 70–85%, respectively. We must highlight that we reported the highest measurements achieved when expert professionals were involved rather than trainee dermatologists. However, they reached around 55–60% accuracy when more classes in the considered taxonomy were included. Accuracy decreased to 40–45% when trainee dermatologists were asked to perform the same task [6,20]. Each mentioned performance evaluation does not belong to the same study, and we must stress that researchers traded off high sensitivity with low specificity in some scenarios. Therefore, the AI-based pipeline proposed in this study met and exceeded the dermatologist-level classification of skin cancer, which does not usually include an automatic anatomical segmentation of the boundaries of the lesions. Undoubtedly, ResNet-3D achieved the best accuracy in the multilabel scenario, attaining peak performance at 92.10% for the MM class (Supplementary Table S3).

## 4. Discussion and Conclusions

The research proposed in this article presented several critical matters. We designed an AI system to assist dermatologists in clustering epidermal tumors, despite the limitation of the small-sized HS dataset. In particular, we researched a robust methodology to develop DL algorithms and cope with small-sized datasets to meet and improve the well-known dermatologist diagnostic performance plateau. Cursed by the absence of large datasets, it took some time for HSI-based applications to become feasible in terms of tasks employing classical RGB or multispectral images. Indeed, the studies considered by the authors of several systematic reviews consisted of databases with significant amounts of data, thus highlighting the diagnostic performance plateau reached. Therefore, classification techniques for HSI often exploit transfer learning and data augmentation to improve classification performances in different research fields [5,6,11]. Algorithms employing HS images usually comprise classical pixel-wise models, such as Support Vector Machines (SVMs), K-Means, and ANNs [12,13]. Despite the fact that the algorithms only work with spectral and not spatial information, their sensitivity and specificity concerning MM and NMSC evaluated through the leave-one-out practice, lie at around 80 and 77%, being recently improved to 87.5 and 100% [14], respectively.

We responded to the demand for AI clinical applications and the lack of computational power to assist it in engineering a handheld instrument equipped with a low-power GPU. The tool should replace the current expensive and time-consuming gold-standard diagnostic procedure to turn the modern DL algorithms into a piece of medical equipment. Recently published articles highlighted that the Food and Drug Administration (FDA) is moving towards approval of AI-based medical devices [19]. It is a crucial turning point considering challenging historical periods, such as the those raised due to the COVID-19 pandemic [23,36]. Not only should AI-based medical instruments aid professionals during challenging times, but they could also be used in frontline emergency clinics, remote places, or the developing world. Concerning skin cancer, we designed and developed a blueprint dermatological device to improve the accessibility of epidermal lesion screening at the global expert level. Expert dermatologist classification accuracy of epidermal lesions usually depends on the number of classes considered. At most, it reaches 85% in a malignant-benign classification scenario. The gold-standard procedure implies clinical and dermoscopic inspection, followed by biopsy and histopathological examination. In other words, the classification accuracy measurement of malignant lesions is biased by the subjective nature of inspection. Indeed, physicians only diagnose lesions already marked as suspicious. We designed a set of CNNs to attain and enhance well-known dermatologist human-level classification performance concerning specificity, sensitivity, and accuracy. Moreover, to the best of the authors’ knowledge, no research was published yet concerning HS skin cancer image segmentation to produce a mask to inform medical doctors about lesion boundaries. Similarly, other studies mainly focused on producing high-end results considering classification scenarios whose clinical applicability is unessential [6,9]. Indeed, not only did we improve the classification taxonomy avoiding poor clustering scenarios where MM is compared against particular lesion types, but we also developed a hyperspectral system containing much more information in terms of RGB, multispectral images, and other spectroscopy techniques. We used artificially intelligent architectures and algorithms to build on the existing literature concerning statistical approaches for spectral signature analysis [37,38,39,40]. Eager to respond to the demand for an AI-based pipeline to assist or replace the present expensive and time-consuming gold-standard procedures [5,9,18], we deployed a semantic segmentation network on a low-power Nvidia Jetson GPU device to be embedded into a portable and handheld medical instrument containing an HS camera. The designed proof-of-concept AI system can classify and segment epidermal lesions in, at most, 1.21 s, and expert professionals could use future implementation in real-world clinical scenarios.

**Figure 6:**
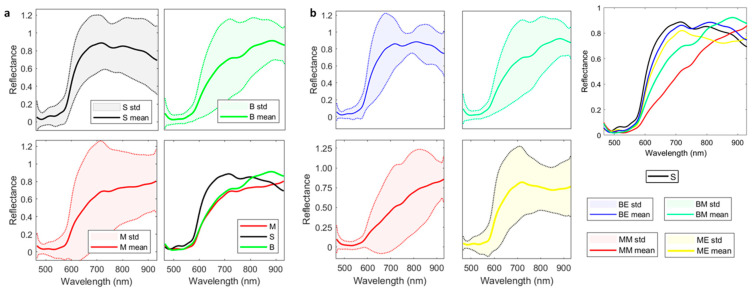
Mean and standard deviation (std) of the spectral signatures of the HS dataset. (**a**) Spectral signatures of skin, Benign and Malignant; (**b**) Spectral signatures of benign epithelial and melanocytic and malignant epithelial and melanocytic. S: Skin, B: Benign; M: Malignant; BE: Benign epithelial; BM: Benign melanocytic; ME: Malignant epithelial; MM: Malignant melanocytic.

Nonetheless, the study exhibits limitations. The main limitation is related to dataset size, which in turn produces others. Indeed, HS imaging is a powerful tool when compared with classical RGB pictures. Chromophores, such as hemoglobin and melanin, characterize skin chemical and spectral properties. They allow the classifying of lesions into different etiologies. HS imaging systems gather skin-reflected and transmitted light into several wavelength ranges on the electromagnetic spectrum, enabling potential skin-lesion differentiation through machine and DL algorithms. Indeed, each pixel contains meaningful information concerning the properties of the object contained in the image. Not only are some lesions in the dataset transitioning from benign towards malignant lesions, but lesions and skin signatures might slightly differ from each other. Moreover, each patient has a unique skin signature which causes the test images to be very different from the training ones, increasing inter-patient variability. Figure 6a represents the spectral signature means and standard deviations of normal skin (S), benign (B), and malignant (M) lesions. In addition, Figure 6b shows the spectral signature mean and standard deviation of each subtype lesion. Therefore, a larger dataset should cope with this problem and allow CNNs to focus more on the meaningful parts of the wavelengths, improving the semantic segmentation results achieved in this work. Indeed, future research should focus more on algorithms which better exploit the huge amount of information contained in a single spectral cube to improve current classification and segmentation performance.

## Data Availability

The datasets generated during the current study are available, under reasonable request, through https://hsidatabase.iuma.ulpgc.es/ (accessed on 31 August 2022). Custom MATLAB codes for HSI data processing and C/CUDA codes for GPU deployment are available under reasonable request.

## Supplementary Materials

The following supporting information can be downloaded at: https://www.mdpi.com/article/10.3390/s22197139/s1, Table S1: HS epidermal dataset description. Table S2: Binary classification performance of the four different approaches for discriminating epidermal lesions using HIS. Table S3: Multilabel classification performance of the four different approaches for discriminating epidermal lesions using HIS. Table S4: Binary segmentation performance of the four different approaches for segmenting epidermal lesions using HSI, including the skin class. Table S5: Multilabel segmentation performance of the four different approaches for segmenting epidermal lesions using HIS. Methods SM: Performance evaluation metrics.
