# Supplementary material for: Neural Networks-Based On-Site Dermatologic Diagnosis through Hyperspectral Epidermal Images
Source: Sensors (Basel). 2022 Sep 21;22(19):7139. doi: 10.3390/s22197139 (PMC9571453; doi:10.3390/s22197139)
Supplement: Supplementary file 1 [file sensors-22-07139-s001.zip › sensors-1921953-supplementary.pdf]

Supplementary Tables

Table S1. HS epidermal dataset description. B: Benign; M: Malignant; MM: Malignant Melanocytic; ME: Malignant Epithelial; BM: Benign Melanocytic; BE: Benign Epithelial.

Capture Date	Subject ID	Capture ID	Binary ID	Multilabel ID	Lesion Location	Diagnosis
20/03/2018	13	C1	M	ME	Chest	Basal cell carcinoma
		C2	B	BM	Stomach	Blue nevus
		C3	B	BM	Stomach	Blue nevus
	14	C1	B	BM	Right Arm	Congenital nevus
	15	C1	B	BM	Back Centre	Nevus
		C2	B	BM	Back Top	Nevus
	16	C1	B	BM	Right Forearm	Nevus
22/03/2018	17	C1	B	BM	Back	Nevus
		C2	B	BM	Back	Nevus
	18	C1	B	BM	Centre Back	Nevus
	20	C2	B	BM	Back	Melanocytic nevus
	21	C1	M	ME	Jaw	Basal cell carcinoma
05/04/2018	23	C1	B	BM	Left Arm	Nevus
	24	C1	B	BM	Left Cheekbone	Solar lentigo
	25	C2	B	BM	Stomach	Nevus
		C3	B	BM	Back	Nevus
	26	C1	B	BM	Right Forearm	Nevus
10/04/2018	27	C1	B	BM	Top Right Back	Nevus
		C2	B	BM	Centre Back	Nevus
		C3	B	BM	Top Left Back	Nevus
		C4	B	BM	Top Left Back	Nevus
	28	C1	B	BM	Neck	Solar lentigo
	29	C1	B	BM	Left Arm	Nevus spilus
		C2	B	BM	Left Arm	Nevus spilus
		C3	B	BM	Left Back	Nevus
	30	C1	B	BM	Right Chest	Nevus
	32	C1	B	BE	Nose	Atypical keratosis
08/08/2018	56	C1	M	ME	Left cheek	Infiltrative basal cell carcinoma
15/02/2019	60	C1	B	BE	Back	Atypical nevus
		C2	B	BM	Left side	Atypical nevus
		C3	B	BE	Left side	Angioma
	61	C1	B	BE	Left leg	Seborrheic keratosis
	62	C1	M	MM	Left eyebrow	Melanoma
22/02/2019	63	C1	B	BM	Stomach	Atypical nevus
	66	C1	M	ME	Left nose	Basal cell carcinoma
	67	C1	M	ME	nose	Carcinoma with Merkel cells
28/02/2019	68	C1	M	MM	Left leg	Melanoma
04/03/2019	69	C1	B	BM	Back	Melanocytic nevus
08/03/2019	71	C1	B	BM	Stomach	Atypical nevus
18/03/2019	74	C1	M	ME	Eye	Basal cell carcinoma
	75	C1	M	ME	Nose/Eye	Basal cell carcinoma
	77	C1	M	ME	Nose	Basal cell carcinoma
25/03/2019	78	C3	B	BE	Ear	Seborrheic keratosis
	79	C1	M	ME	Brow	Basal cell carcinoma
	80	C1	M	ME	Head	Epidermoid carcinoma
	81	C1	M	MM	Abdomen	Melanoma
28/03/2019	82	C1	M	MM	Right arm	Melanoma
01/04/2019	83	C1	B	BE	Nose	Seborrheic keratosis
05/04/2019	86	C1	B	BM	Chest	Atypical nevus
		C2	B	BM	Stomach	Atypical nevus
		C3	B	BM	Back	Atypical nevus
		C4	B	BM	Back	Atypical nevus
	87	C1	M	MM	Left cheek	Malignant lentigo
08/04/2019	88	C1	M	ME	Right shoulder	Basal cell carcinoma
	89	C1	M	ME	Left ear	Basal cell carcinoma

Capture Date	Subject ID	Capture ID	Binary ID	Multilabel ID	Lesion Location	Diagnosis
	90	C1	M	ME	Head	Basal cell carcinoma
22/04/2019	91	C1	M	ME	Face	Basal cell carcinoma with infiltration
	92	C1	M	ME	Neck	Epidermoid carcinoma
23/04/2019	94	C1	M	MM	Back	Melanoma Clark level III-IV
26/04/2019	95	C1	M	MM	Buttock	Melanoma
06/04/2019	96	C1	M	MM	Right leg	Melanoma
29/04/2019	97	C1	M	MM	Chest	Melanoma Clark level II
	98	C1	M	MM	Back	Dysplastic Nevus
	99	C1	M	ME	Face	Basal cell carcinoma
06/05/2019	100	C1	M	ME	Neck	Basal cell carcinoma
	101	C1	M	ME	Right hand	Basal cell carcinoma
07/05/2019	102	C1	M	MM	Back	Melanoma Clark level IV
17/05/2019	103	C1	B	BM	Right forearm	Blue nevus
20/05/2019	104	C1	M	ME	Nasal wing	Basal cell carcinoma
24/05/2019	106	C1	M	MM	Nose	Malignant lentigo
27/05/2019	107	C1	B	BM	Left arm	Blue nevus
	109	C1	M	ME	Back left hand	Epidermoid carcinoma
03/06/2019	110	C1	M	ME	Head	Basal cell carcinoma
	112	C1	M	ME	Helix	Basal cell carcinoma
	113	C1	M	ME	Nose	Epidermoid carcinoma
10/06/2019	116	C1	M	ME	Face	Basal cell carcinoma

Table S2. Binary classification performance of the four different approaches for discriminating epidermal lesions using HSI. AUC: Area Under Curve; B: Benign; M: Malignant.

	Class ID	ResNet18	ResNet50	ResNet101	ResNet3D
**Accuracy (%)**	-	85.53	85.53	86.84	**88.16**
**Sensitivity (%)**	-	82.50	82.50	85.00	**87.50**
**Specificity (%)**	-	**88.89**	**88.89**	**88.89**	**88.89**
**Precision (%)**	B	89.19	89.19	89.47	**89.74**
	M	82.05	82.05	84.21	**86.49**
**F1-Score (%)**	B	85.71	85.71	87.18	**88.61**
	M	85.33	85.33	86.49	**87.67**
**AUC**	B	0.93	**0.94**	0.93	0.91

Table S3. Multilabel classification performance of the four different approaches for discriminating epidermal lesions using HSI. AUC: Area Under Curve; MM: Malignant Melanocytic; ME: Malignant Epithelial; BM: Benign Melanocytic; BE: Benign Epithelial.

	Class ID	ResNet18	ResNet50	ResNet101	ResNet3D
**Accuracy (%)**	MM	90.79	92.11	**93.42**	92.11
	ME	84.21	**85.53**	**85.53**	**85.53**
	BE	85.53	**86.84**	84.21	**86.84**
	BM	84.21	88.16	86.84	**90.79**
**Sensitivity (%)**	MM	**50.00**	**50.00**	**50.00**	**50.00**
	ME	85.29	85.29	85.29	**88.24**
	BE	**79.17**	**79.17**	**79.17**	**79.17**
	BM	58.33	**75.00**	58.33	**75.00**
**Specificity (%)**	MM	**98.57**	**98.57**	**98.57**	**98.57**
	ME	83.33	**85.71**	**85.71**	83.33
	BE	88.46	**90.38**	86.54	**90.38**
	BM	89.06	90.63	92.19	**93.75**
**Precision (%)**	MM	**83.33**	**83.33**	**83.33**	62.50
	ME	80.56	**82.86**	**82.86**	81.08
	BE	76.00	**79.17**	73.08	**79.17**
	BM	50.00	60.00	58.33	**69.23**

**F1-Score (%)**	MM	**62.50**	**62.50**	**62.50**	55.56
	ME	82.86	84.06	84.06	**84.51**
	BE	77.55	**79.17**	76.00	**79.17**
	BM	53.85	66.67	58.33	**72.00**
**AUC**	MM	0.60	**0.70**	0.57	0.46
	ME	0.15	0.12	0.14	**0.16**
	BE	0.47	0.55	**0.56**	0.46
	BM	0.35	**0.39**	0.37	0.35

Table S4. Binary segmentation performance of the four different approaches for segmenting epidermal lesions using HSI, including the skin class. IOU: Intersection Over Union; MBFS: Mean Border F-Score; AUC: Area Under Curve; S: Skin; M: Malignant; B: Benign; DL: DeepLab; RN: ResNet.

	**Class ID**	**U-Net**	**U-Net++**	**DLV3+ RN18**	**DLV3+ RN50**
**DICE**	S	0.94	**0.95**	0.41	0.94
	B	0.58	**0.73**	0.19	0.62
	M	0.54	**0.68**	0.16	0.58
**IOU**	S	0.88	**0.90**	0.25	0.88
	B	0.41	**0.58**	0.11	0.45
	M	0.37	**0.51**	0.09	0.41
**MBFS**	S	0.76	**0.77**	0.40	0.68
	B	0.16	**0.26**	0.05	0.15
	M	0.09	**0.16**	0.05	0.11
**Accuracy (%)**	S	**96.08**	95.39	27.00	94.21
	B	52.36	**71.20**	51.99	58.98
	M	48.56	**67.24**	27.86	59.20
**Sensitivity (%)**	S	**96.08**	95.39	27.00	94.21
	B	52.36	**71.20**	51.99	58.98
	M	48.56	**67.24**	27.86	59.20
**Specificity (%)**	S	65.97	**79.46**	77.99	76.46
	B	96.75	**97.29**	53.36	96.35
	M	96.04	**96.16**	73.26	94.35
**Precision (%)**	S	91.09	**94.39**	81.63	93.55
	B	65.77	**75.81**	11.74	65.82
	M	60.17	**68.36**	11.38	56.38
**FI-Score (%)**	S	93.52	**94.89**	40.58	93.88
	B	58.30	**73.43**	19.15	62.21
	M	53.75	**67.80**	16.16	57.75
**AUC**	S	**0.75**	0.70	0.55	0.72
	B	**0.74**	0.63	0.56	0.71
	M	**0.64**	0.56	0.51	0.61

Table S5. Multilabel segmentation performance of the four different approaches for segmenting epidermal lesions using HSI. IOU: Intersection Over Union; MBFS: Mean Border F-Score; AUC: Area Under Curve; S: Skin; MM: Malignant Melanocytic, ME: Malignant Epithelial, BM: Benign Melanocytic, BE: Benign Epithelial, DL: DeepLab; RN: ResNet.

	**Class ID**	**U-Net**	**U-Net++**	**DLV3+ RN18**	**DLV3+ RN50**
**IOU**	S	0.88	**0.91**	0.36	0.89
	MM	0.34	**0.56**	0.02	0.53
	ME	0.17	**0.34**	0.02	0.26
	BE	0.01	**0.17**	0.01	0.04
	BM	0.37	**0.64**	0.02	0.61
**DICE**	S	0.93	**0.95**	0.53	0.94
	MM	0.51	**0.72**	0.04	0.69
	ME	0.29	**0.51**	0.05	0.42

	BE	0.02	**0.29**	0.02	0.08
	BM	0.54	**0.78**	0.03	0.76
**MBFS**	S	0.76	**0.77**	0.62	0.71
	MM	0.09	**0.25**	0.02	0.19
	ME	0.07	**0.09**	0.03	0.08
	BE	0.05	**0.10**	0.04	0.09
	BM	0.15	**0.30**	0.02	0.21
**Accuracy (%)**	S	**96.30**	95.40	39.13	94.92
	MM	48.56	71.27	3.52	**72.73**
	ME	21.22	**48.53**	3.82	37.31
	BE	1.11	19.80	**57.58**	5.66
	BM	53.67	81.34	1.78	**75.63**
**Sensitivity (%)**	S	**96.30**	95.40	39.13	94.92
	MM	48.56	71.27	3.52	**72.73**
	ME	21.22	**48.53**	3.82	37.31
	BE	1.11	19.8	**57.58**	5.66
	BM	53.67	**81.34**	1.78	75.63
**Specificity (%)**	S	64.29	**80.82**	64.08	74.00
	MM	97.91	**98.71**	96.67	98.20
	ME	**98.34**	97.15	96.18	97.19
	BE	99.74	**99.81**	47.40	99.63
	BM	95.27	97.08	**98.25**	97.50
**Precision (%)**	S	90.71	**94.74**	79.78	92.97
	MM	53.11	**72.90**	4.91	66.32
	ME	46.26	**53.45**	6.32	47.25
	BE	4.65	**54.05**	1.24	14.95
	BM	54.45	74.57	9.67	**76.09**
**F1-Score (%)**	S	93.42	**95.07**	52.5	93.94
	MM	50.73	**72.08**	4.10	69.38
	ME	29.09	**50.87**	4.76	41.70
	BE	1.80	**28.98**	2.43	8.21
	BM	54.06	**77.81**	3.01	75.86
**AUC**	S	**0.74**	0.71	0.53	0.71
	MM	**0.61**	0.40	0.54	0.59
	ME	**0.71**	0.62	0.52	0.66
	BE	**0.74**	0.69	0.50	0.65
	BM	**0.71**	0.67	0.50	0.64

Supplementary Methods

Performance evaluation metrics

The occurrence of true-positive (TP), true-negative (TN), false-positive (FP) and false-negative (FN) values were computed to evaluate the DL architectures performance. Concerning the semantic segmentation task, the pixel-based occurrences were assessed. The assessment outcomes were exploited to compute the following metrics: *accuracy* (Eq. S1), *sensitivity* (Eq. S2), *specificity* (Eq. S3), *precision* (Eq. S4), *Receiver Operating Characteristic Area Under the Curve (AUC), and F1-Score (Eq. S5)*. For the segmentation task we also computed the *Mean Boundary-F1 Score (MBFS)*, namely the average F1 Score computed on the lesions' boundaries, the *Intersection Over Union (IOU, Eq. S6)*, and the *DICE coefficient (Eq. S7).*

$$Accuracy = \frac{TP + TN}{P + N} \quad \text{(S1)}$$

$$Sensitivity = \frac{TP}{TP + FN} \quad \text{(S2)}$$

$$Specificity = \frac{TN}{TN + FP} \quad \text{(S3)}$$

$$Precision = PPV = \frac{TP}{TP + FP} \tag{S4}$$

$$F1\ Score = \frac{2\ \cdot Precision \cdot Recall}{Precision + Recall} \tag{S5}$$

$$IOU = \frac{|A\ \cap\ B|}{|A\ \cup\ B|} \tag{S6}$$

$$DICE = \frac{2 \cdot |A\ \cap\ B|}{|A\ \cup\ B|} \tag{S7}$$
